# Fiber-Optic Sensor Spectrum Noise Reduction Based on a Generative Adversarial Network

**DOI:** 10.3390/s24227127

**Published:** 2024-11-06

**Authors:** Yujie Lu, Qingbin Du, Ruijia Zhang, Bo Wang, Zigeng Liu, Qizhe Tang, Pan Dai, Xiangxiang Fan, Chun Huang

**Affiliations:** 1 School of Information Engineering, Huzhou University, Huzhou 313000, China; 2024388110@stu.zjhu.edu.cn (Q.D.); 2021082526@stu.zjhu.edu.cn (R.Z.); 02903@zjhu.edu.cn (Q.T.); 02627@zjhu.edu.cn (P.D.); 02502@zjhu.edu.cn (X.F.); 02874@zjhu.edu.cn (C.H.); 2 Acoustic Science and Technology Laboratory, Harbin Engineering University, Harbin 150001, China; s323057038@hrbeu.edu.cn

**Keywords:** fiber-optic sensing, generative adversarial network, noise reduction, signal processing

## Abstract

In the field of fiber-optic sensing, effectively reducing the noise of sensing spectra and achieving a high signal-to-noise ratio (SNR) has consistently been a focal point of research. This study proposes a deep-learning-based denoising method for fiber-optic sensors, which involves pre-processing the sensor spectrum into a 2D image and training with a cycle-consistent generative adversarial network (Cycle-GAN) model. The pre-trained algorithm demonstrates the ability to effectively denoise various spectrum types and noise profiles. This study evaluates the denoising performance of simulated spectra obtained from four different types of fiber-optic sensors: fiber Fabry–Perot interferometer (FPI), regular fiber Bragg grating (FBG), chirped FBG, and FBG pair. Compared to traditional denoising algorithms such as wavelet transform (WT) and empirical mode decomposition (EMD), the proposed method achieves an SNR improvement of up to 13.71 dB, an RMSE that is up to three times smaller, and a minimum correlation coefficient ($R^{2}$) of no less than 99.70% with the original high-SNR signals. Additionally, the proposed algorithm was tested for multimode noise reduction, demonstrating an excellent linearity in temperature response with a $R^{2}$ of 99.95% for its linear fitting and 99.74% for the temperature response obtained from single-mode fiber sensors. The proposed denoising approach effectively reduces the impact of various noises from the sensing system, enhancing the practicality of fiber-optic sensing, especially for specialized fiber applications in research and industrial domains.

## 1. Introduction

The development of fiber-optic sensors has rapidly grown in recent decades due to the advantages of these devices, such as small size, light weight, low cost, high sensing sensitivity, and ability to operate in harsh environments [1,2,3]. Fiber-optic sensors have become a viable alternative to traditional electronic sensors in many research and industrial areas because of their outstanding chemical corrosion immunity and electromagnetic insensitivity [4,5]. In many application scenarios of fiber-optic sensing, the signal-to-noise ratio (SNR) is an important means of validating the sensor performance. A fiber-optic sensing system usually includes optical and electronic modules, both of which can introduce noise during processes such as light transmission, sensing, and signal processing [6,7,8]. Furthermore, fiber-optic sensors fabricated from specialty materials, such as single-crystal sapphire fiber and plastic fiber, are being used in special scenarios like high-temperature, corrosive environments or when there are lightweight requirements [9,10]. Although these fibers have outstanding physical and chemical properties, most of them are unable to operate in the single-mode transmission state, resulting in a significant effect of the mode noise. To obtain a higher SNR in fiber-optic sensing systems, scientists have made efforts to reduce noise. One of the most common methods is using a filter algorithm, such as a digital bandpass filter or wavelet-based noise reduction [11,12,13]. Many researchers have also explored methods of improving the fiber-optical sensing system design, for example, using a multi-channel system to build a noise signal compensation schematic; reducing the fiber diameter by acid-etching the fiber; or directly writing the radial waveguide around the fiber center to generate single-mode transmission in the multimode fiber [14,15,16]. Most of the noise reduction approaches require additional technical or denoising procedures in fiber processing to achieve a high SNR signal. The denoising algorithms also have limitations in terms of frequency and type of noise. This has an adverse impact on the reliability and practicality of fiber-optic sensors, consequently hindering their widespread adoption.

In recent years, deep learning has become one of the most prominent technologies in both research and industrial domains. By leveraging different types of deep learning algorithms, researchers have achieved remarkable progress in areas such as computer vision, natural language processing, and image processing [17,18,19]. Deep learning also shows exceptional application potential in application areas such as medical, financial, agriculture, and industrial manufacturing [20,21,22,23]. In the optical sensing field, some researchers have used deep learning algorithms to enhance sensing signals and classify sensing events. For instance, in 2021, Safati proposed a shape sensing and position estimation method of a surgical continuum manipulator [24], utilizing data collected from fiber-optic sensors and processed by a machine learning model. In 2024, Roodsari introduced a high spatial resolution fiber shape sensing method based on the deep learning model [25].

In this paper, we propose an algorithm to reduce the different types of spectrum noises from fiber-optic sensors based on a cycle-consistent generative adversarial network (Cycle-GAN) [26]. Compared to the ordinary noise reduction methods, the Cycle-GAN-based denoising algorithm demonstrates superior generalizability and adaptability to different fiber sensor types and spectrum characteristics. It also exhibits a better denoising accuracy and real-time processing ability compared to the traditional sensing noise reduction algorithm. Ultimately, this denoising algorithm not only offers a novel approach to noise reduction on the fiber-optic sensors but also enhances the current fiber-optic sensing system in research and industrial applications.

## 2. Noise Reduction Algorithm for Fiber-Optic Sensor Spectra

The denoising procedure can be divided into two main steps: spectrum training and spectrum denoising training. In the spectrum training, the noise and clear fiber sensor spectra are input to the computer for training. This training is performed individually based on different spectrum characteristics and noise types. In this part, the computer is trained to identify the required spectrum and discard all the noise that does not belong to the high-SNR sample spectrum.

### 2.1. Spectrum Pre-Processing

**Figure 1:**
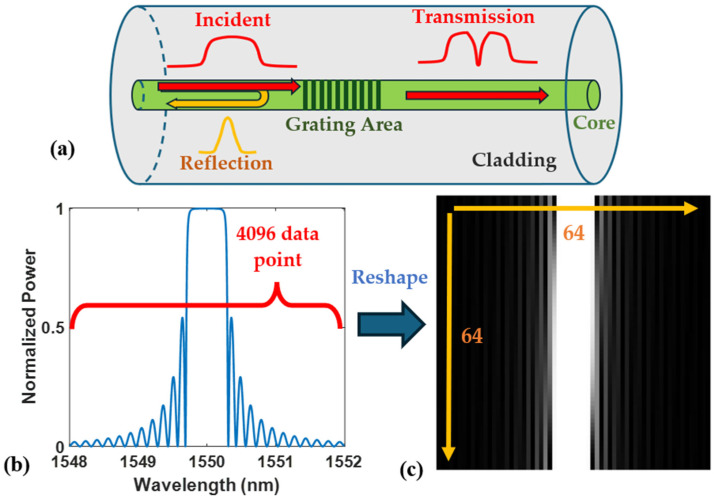
(**a**) The structure of a regular FBG; (**b**) a normalized FBG spectrum obtained from simulation, and (**c**) its reshaped 2D image.

Fiber-optical sensor spectra consist of one-dimensional data, whereas image processing algorithms typically operate on two-dimensional (2D) or higher-dimensional datasets. Although an original spectrum picture can be employed as the input of a Cycle-GAN, the picture is too large for the training, takes too much time, and requires too many resources. Hence, 1D spectrum data should be resized into a 2D picture before training and transforming. In our demonstration, all the data were pre-processed using MATLAB, including the simulation and experimental data. Figure 1a illustrates a regular high-reflection fiber Bragg grating (FBG) structure, where periodic refractive index (RI) modulation is applied along the silica fiber axis. As the incident light propagates to the grating area, it generates partial reflection across a specific wavelength range. The FBG is simulated by using the 2×2 transfer matrix T of the uniform FBG, as described in the following function [27]:

(1) $$T=\left[\begin{array}{cc} T_{11} & T_{12}\\ T_{21} & T_{22} \end{array}\right]=\left[\begin{array}{cc} \cosh\left(\gamma l\right)-i\frac{∆\beta }{\gamma }\sinh\left(\gamma l\right) & -\frac{\kappa }{\gamma }\sinh\left(\gamma l\right)\\ i\frac{\kappa }{\gamma }\sinh\left(\gamma l\right) & \cosh\left(\gamma l_{i}\right)+i\frac{\Delta \beta }{\gamma }\sinh\left(\gamma l\right) \end{array}\right]$$

where γ denotes the self-coupling coefficient, and κ is the coupling coefficient as a function of wavelength λ and RI modulation. The l and ∆β correspond to the grating length and wave vector detuning parameter, respectively. Both γ and κ are the functions of l and λ, while ∆β is related to the central Bragg wavelength, $\lambda_{B}$, which is considered as a constant for a given FBG. As a result, for each FBG with distinct central Bragg wavelength, the total reflectivity can be expressed as the following function:

(2) $$R(\lambda ,l)={\left|\frac{T_{21}}{T_{11}}\right|}^{2}$$

Figure 1b presents a spectrum of a simulated high-reflectivity FBG obtained using the transfer matrix method. The spectrum comprises 4096 data points within the wavelength range from 1548 to 1552 nm.

In this paper, three fiber-grating-based sensor types and a fiber Fabry–Perot Interferometer (FPI) sensor are presented to demonstrate the denoising capability of our algorithm. The numerical approaching of the FPI with its total reflectivity $R_{tot}$ can be described as a function of wavelength λ as:

(3) $$R_{tot}=\frac{2R+2R\cos\left(\frac{4\pi nl}{\lambda }\right)}{1+R^{2}+2R\cos\left(\frac{4\pi nl}{\lambda }\right)}$$

where R represents the reflectivity at the interface between the fiber core and FP cavity medium, while n and l are the RI and cavity length of the FP cavity, respectively. For a specific FPI with a fixed FP cavity medium, both R and n are constant. The spectrum can be modified by varying the cavity length.

It should be considered that the chosen data range may vary for different types of spectra. For example, the FPI spectrum interception range is broader than the FBG-related spectrum, which is generally concentrated around the central grating wavelength. Since the original data are recorded using decibel or linear scales, they cannot be directly transferred to the picture data. Thus, normalization should be performed before training or transferring. On the other hand, the different ranges of the input data affect the evaluation and discrimination procedures during the training, generating an error that reduces the output accuracy. The normalization optical intensity employed in this study, denoted by $I_{norm}$, can be expressed as follows:

(4) $$I_{norm}=\frac{I-I_{min}}{I_{max}-I_{min}}$$

where I, $I_{max}$, and $I_{min}$ are the original, maximum, and minimum power intensities along the wavelength, respectively. The above equation maps the original intensity to the range between 0 and 1 while maintaining the original data relations. The normalized data were reshaped to a 2D grayscale image. For each pixel on the 2D reshaped picture, the grayscale-level values vary from 0 to 255, containing sufficient information for further data processing while keeping the required spectrum and noise for reconstructing the spectrum after noise reduction. Hence, the FBG spectrum was reshaped to a 64×64 grayscale picture as shown in Figure 1c. In the following tests, the simulation test group included 4096 data points, while the experimental data only contained 1024 data points due to the interrogator limitation.

### 2.2. Spectrum Denoising Training

**Figure 2:**
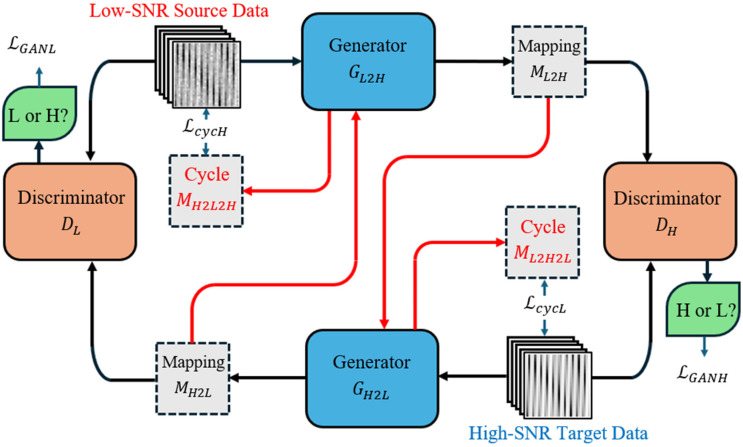
Cycle-GAN denoising flow chart for a fiber-optic sensor spectrum.

The Cycle-GAN-based denoising algorithm needs unsupervised training to recognize the signal and noise prior to its application in denoising the fiber-optical sensor spectrum. The training procedure requires two classes of dataset, one containing the source low-SNR spectrum reshaped images with a high noise level and another containing the high-SNR spectrum reshaped images, called the original and target data domains and denoted by L and H, respectively. The overall training flow is shown in Figure 2.

The denoising algorithm operates through two end-to-end generators, $G_{L2H}$ and $G_{H2L}$, along with two discriminators, $D_{L}$ and $D_{H}$. Each generator contains four main components: three convolutional (Conv) layers, a residual neural network consisting of nine residual blocks (9-block ResNet), two deconvolutional (Deconv) layers, and a final Conv layer. The initial three Conv layers are used to extract low-level features from the input image while reducing its spatial dimensions and increasing its depth. The 9-block ResNet serves as the core of the generator within the Cycle-GAN model, playing a crucial role in residual learning and feature extracting, thereby enhancing the generator’s performance and stability. After passing through the residual blocks, the data are sent to the Deconv layers for up-sampling to a higher resolution and transforming back to the original image dimensions of the target domain. Finally, the additional Conv layer reduces the output image depth to match the desired channel configuration.

The discriminator structure in the Cycle-GAN model is relatively simple, containing three Conv layers, a batch normalization layer, a Leaky ReLU activation function, and an output layer. The first three Conv layers function similarly to those in the generators, extracting essential features from the images. The normalization layer stabilizes the training process by normalizing the outputs of each Conv layer, helping to reduce internal covariate shift and improve convergence speed. The Leaky ReLU activation function is applied after each normalization layer to enhance the gradient flow. The final layer is a single Conv layer that outputs a feature map with a single channel, facilitating classification decision regarding the input image.

These generators and discriminators engage in a competitive process throughout the training phase. The generators aim to generate fake examples that resemble data from the opposing domain. In contrast, the discriminators are attempting to discern whether the input data are authentic or fabricated. In our denoising model, the generator $G_{L2H}$ is used to transform the low-SNR source data into the same data as in the high-SNR target domain. The generated data, $M_{L2H}$, which represents the mapping from the low-SNR domain to the high-SNR domain, are sent to the discriminator $D_{H}$ for classification. $D_{H}$ contains both the real data from the high-SNR domain and the fake data generated by $G_{L2H}$, and its function is to distinguish between them. The incurred loss during the generation and discrimination can be described as the adversarial loss with the following function [28]:

(5) $$L_{GANH}\left(G_{L2H},D_{H},L,H\right)=E_{H\sim p\left(H\right)}\left[\log D_{H}\left(H\right)\right]+E_{L\sim p\left(L\right)}\left[1-\log D_{H}\left(L\right)\right],$$

where $E_{H\sim p\left(H\right)}$ and $E_{L\sim p\left(L\right)}$ represent the expectations of data distribution for the high-SNR and low-SNR spectra. $D_{H}\left(H\right)$ and $D_{H}\left(L\right)$ denote the data from the real high-SNR domain and the generated data, respectively.

On the other hand, the generated data $M_{L2H}$ are sent to another generator $G_{L2H}$ to map the data back to the low-SNR domain. The output data $M_{L2H2L}$ are compared to the original low-SNR data to confirm whether $M_{L2H}$ remains consistent during the process. The loss in this procedure can be expressed as the function below:

(6) $$L_{cycL}\left(G_{L2H},G_{H2L}\right)=E_{L\sim p\left(L\right)}\left[\left\|G_{H2L}\left(G_{L2H}\left(data_{L}\right)\right)-data_{L}\right\|\right],$$

In this function, the output $L_{cycL}$ is called cycle consistency loss. $data_{L}$ represents the data from the low-SNR domain, and $G_{H2L}\left(G_{L2H}\left(data_{L}\right)\right)$ denotes the Low→High→Low transform procedure across the two generators. The entire training process includes another opposite path as described before. Thus, the opposite loop also generates the adversarial loss and cycle consistency loss as below:

(7) $$L_{GANL}\left(G_{H2L},D_{L},H,L\right)=E_{L\sim p\left(L\right)}\left[\log D_{L}\left(L\right)\right]+E_{H\sim p\left(H\right)}\left[1-\log D_{L}\left(H\right)\right],$$

(8) $$L_{cycH}\left(G_{H2L},G_{L2H}\right)=E_{H\sim p\left(H\right)}\left[\left\|G_{L2H}\left(G_{H2L}\left(data_{H}\right)\right)-data_{H}\right\|\right],$$

Ultimately, the overall loss function used to evaluate the entire transformation effect throughout the training cycle can be expressed by the following equation:

(9) $$L\left(G_{L2H},G_{H2L},D_{L},D_{H}\right)=L_{GANH}\left(G_{L2H},D_{H},L,H\right)+L_{GANL}\left(G_{H2L},D_{L},H,L\right)+\;\Lambda L_{cyc}\left(G_{L2H},G_{H2L}\right),$$

where $L_{cyc}\left(G_{L2H},G_{H2L}\right)$ is the sum of the cycle consistency losses from two directions, and Λ represents the relative importance value that balances the weights of $L_{GAN}$ and $L_{cyc}$. In the adversarial process, the overall loss is used to evaluate the accuracy of generation, and the aim of the training program is to minimize the loss. The training cycle is repeated thousands of times to self-modify the denoising parameters, ultimately leading to a zero-sum game where the discriminators can only make a 50% guess.

The training iteratively enhances the reliability of the fake output spectrum data generated by $G_{L2H}$, ultimately achieving a high-SNR spectrum without additional noise. Notably, the training and denoising processes are based on the characteristics of the images. This procedure involves feature extraction from the low-SNR spectrum images and reconstructing them with the features from the high-SNR data domain. Consequently, critical information essential for further analysis, such as the free spectrum range, wavelength, or spectrum shape, is preserved. As a result, the training process empowers the denoising model with the capability to effectively eliminate noise from its corresponding spectrum type. Depending on the computer configuration, the denoising procedure utilizing a pre-trained Cycle-GAN model only takes a few seconds.

## 3. Training Data Acquisition and Experimental Setup

To validate the effectiveness of the Cycle-GAN-based denoising algorithm, we conducted tests on both numerically simulated and experimentally measured sensing spectra. Four different types of typical fiber-optic sensor spectra were simulated for training and testing using the Cycle-GAN-based denoising algorithm. Additionally, a pair of single-mode fiber (SMF) FPI and multimode fiber (MMF) FPI sensors was fabricated to generate sensing spectra for modal noise reduction training and testing. By applying our denoising algorithm, we were able to assess the algorithm’s capability and efficiency in real test scenarios.

### 3.1. Simulated Fiber-Optic Sensor Spectra

**Figure 3:**
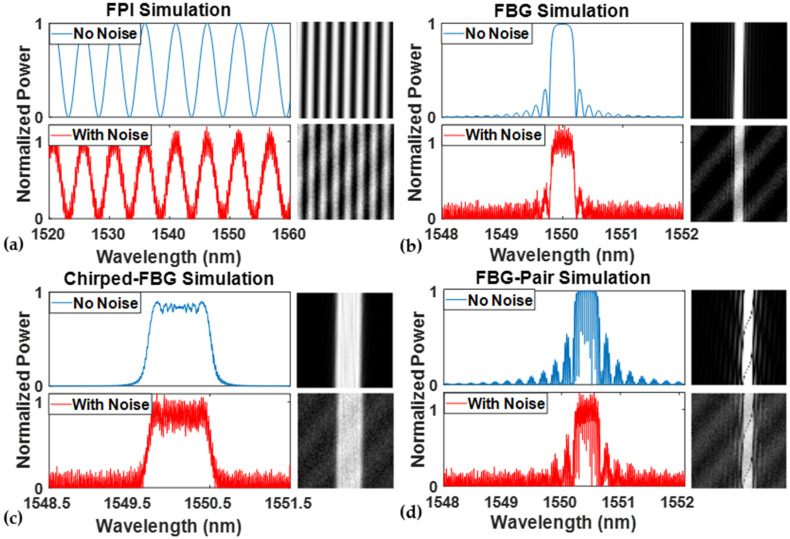
Simulation of high-SNR and noise-added spectra of (**a**) regular low-finesse FPI; (**b**) regular FBG; (**c**) chirped FBG; and (**d**) FBG pair sensors. The corresponding reshape gray-level image is on the right side of each spectrum.

The denoising training was performed to verify the noise reduction algorithm’s feasibility, and the denoising ability was evaluated using different fiber sensor spectra from simulations. Four simulated spectra from different fiber-optic sensors were introduced, as shown in Figure 3. Figure 3a,b show the spectra from a simulated regular low-finesse FPI and a regular FBG sensor. In contrast, Figure 3c,d show the simulation spectra from a chirped FBG and an FBG pair sensor. The three fiber-grating-based sensor types were simulated by the same transfer matrix method as detailed in Equations (1) and (2) with minor modifications. For each sensor type shown in Figure 3, the top picture is the high-SNR spectrum from the original simulation model. Random and periodic noises were applied to each original sensor signal to obtain the low-SNR spectrum. Their pre-processed 2D images were located at the right side of each spectrum. Regular FPI and FBG sensors are the two commonly used sensors in the fiber-optical sensing field. The denoising performance can be employed to evaluate the efficiency of periodic and aperiodic spectra. The chirped FBG and FBG pair have particular spectrum characteristics: the chirped FBG has a relatively flat central band, while the FBG pair has a denser interference fringe superimposed on the regular FBG spectrum. The study of these spectra demonstrates the efficiency and generality of the Cycle-GAN-based denoising algorithm.

For the training of the denoising algorithm, 2000 pairs of high-SNR and low-SNR spectrum data were generated for each type of simulated sensor spectra. The number of the spectrum data point is 4096, leading to a 64×64 reshaped image for further training. During the training process of each sensor type, the 2000 original high-SNR spectra were set as the target training data, while the low-SNR spectra were employed as the source data. The spectra were repeatedly trained in the Cycle-GAN model until the discriminator could not distinguish between the data generated form the low-SNR spectrum and the high-SNR target data. The training for each sensor type extended up to 3000 epochs, with the loss values from Equations (4)–(8) recorded for each epoch. These loss values, particularly the cycle-consistency loss and discriminator loss, are critical parameters for evaluating the model’s accuracy in transforming low-SNR to high-SNR spectra. To enhance the training efficiency and improve the denoising performance, various training parameters, including batch size and image load size, were also optimized.

### 3.2. Experiment Setup for Modal Noise Reduction

**Figure 4:**
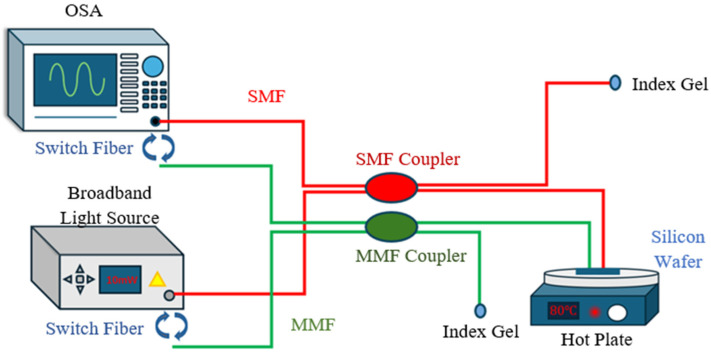
Setup of the FPI sensor’s modal noise reduction.

Unlike typical electronic devices, fiber-optic devices are subject to modal noise, which can significantly degrade the optical signal accuracy. This kind of noise commonly exists when a multimode fiber is used, and sometimes it can prevent a clear transmission signal from being obtained. The constructed setup shown in Figure 4 generates the training data for the denoising algorithm. The broadband light source is the illumination source for the entire system, providing a flat light signal for the sensor spectrum. The light emitted from the source propagates through the optical fiber and is split by a 2×2 coupler, where only one of the outputs is employed for connecting the sensor. Index gels were applied to the unused output end of the coupler to remove the effect of the additional end face reflection. Optical UV glue (NOA 61) is used to bond the fiber end directly to a silicon wafer having a thickness of 80 μm. This silicon wafer serves as the FP cavity for obtaining the interference sensing spectrum. The reflection spectrum travels back through the coupler and is received by the optical spectrum analyzer (OSA) for the acquisition of sensing data.

Two types of fibers and couplers were utilized during the test: the regular SMF and the 62.5/125 MMF. Both the SMF and MMF sensing tests shared the same experimental setup as shown above. The two test sets were processed alternately by switching the transmission fiber and the corresponding coupler. The SMF and MMF were affixed to the same silicon wafer to obtain spectra with similar free spectrum range (FSR). To ensure a consistent and secure attachment of both fibers to the silicon wafer, UV glue was applied on the fiber ends. In both the SMF and MMF test sets, the spectra were continuously recorded while the temperature of the hot plate was increased from room temperature to 100 ℃ and then allowed to naturally cool down. The temperature didn’t increase over 100 ℃ due to the limitation of the UV glue. This temperature adjustment process was repeated several times to record 1000 spectra of each fiber test set for further training purposes.

**Figure 5:**
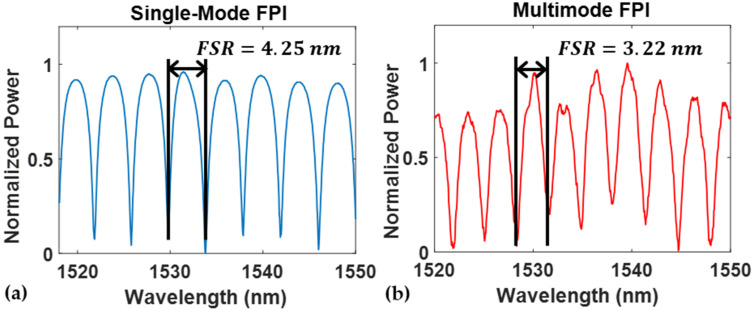
Silicon wafer FPI spectrum with (**a**) SMF and (**b**) MMF as the lead-in fiber.

Figure 5 presents the original spectrum obtained from the SMF-FPI and MMF-FPI experimental setup. Although the SMF and MMF test sets employed the same silicon wafer, the FSR from the two test sets exhibited differences. With the 80 μm silicon wafer, the FSR from the SMF test set is approximately 4.25 nm, while the MMF contributes an FSR of about 3.22 nm, corresponding to a 107 μm silicon FP cavity length. This difference may be attributed to the larger core diameter and output angular distribution of the MMF. However, the denoising algorithm for the FPI sensor is independent of frequency. These spectra were processed on another computer for reshaping and denoising training. The spectrum data length for both SMF and MMF sensors was 2501 due to the OSA and light source limitation. Consequently, 1024 data points were intercepted from the original spectrum to form a 32 × 32 image for further training. After 3000 epochs of training in the Cycle-GAN-based denoising model, the model with the lowest overall losses was selected, resulting in the successful denoising of the MMF spectrum.

## 4. Denoising Results and Discussion

Both the simulation and experimental spectra were pre-processed and subsequently fed into the Cycle-GAN model for training. After sufficient training with thousands of data points and epochs, each type of sensor spectrum had its own specific denoising parameters and was ready for denoising. We applied the algorithm to different simulated spectra as mentioned above to evaluate the denoising capabilities of Cycle-GAN-based denoising for various spectra. Two classic spectrum denoising algorithms were also utilized to compare their denoising results with ours. Additionally, using the spectra obtained in the modal noise reduction experiment setup as shown in Figure 2, we conducted a series of temperature response tests to validate the practical applicability of the denoising algorithm.

**Figure 6:**
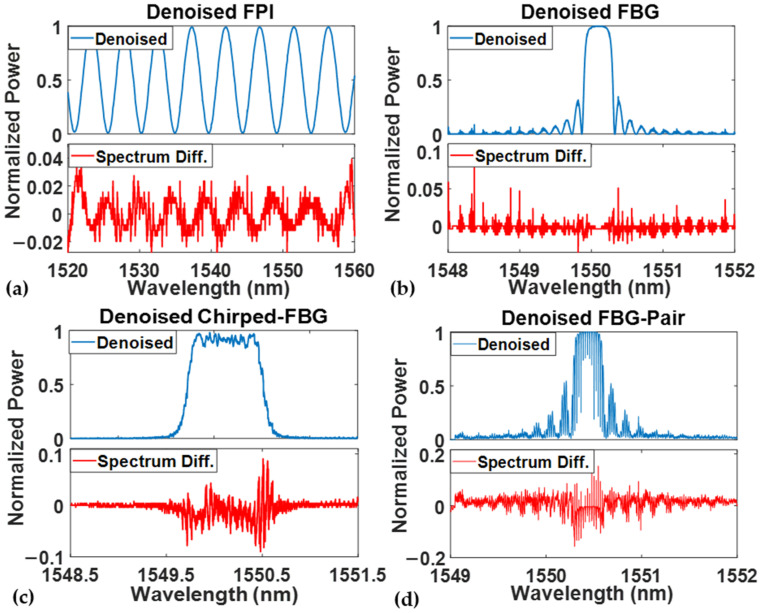
Cycle-GAN-based algorithm denoising results of (**a**) regular low-finesse FPI; (**b**) regular FBG; (**c**) chirped FBG; and (**d**) FBG pair sensors. For each sensor, the top half is the denoised spectrum and the bottom half is the difference from the high-SNR spectrum.

The denoising results of the four simulation spectra are shown in Figure 6. The denoised spectra and their remaining noise difference are presented for each sensor type. In the four simulated sensor denoising tests, the low-finesse FPI has the slightest difference between the original high-SNR and the denoised spectrum. Once the spectrum detail increases, the remaining spectrum difference level grows, while the regular and chirped FBG sensors have similar spectrum differences. In the FBG spectrum, more remaining noises are located at the side-band peaks, while most of the noises remain at the central range of the flat peak in the chirped FBG test. The sharp interference valleys in the FBG pair have different depths due to the wavelength resolution of the simulated spectrum, causing a relatively high spectrum difference in the denoising spectrum generation. However, the denoised spectrum still shows ideal performance and is similar to the original high-SNR spectrum. The denoising results can be improved by increasing the data resolution. Another 100 high-SNR spectra were simulated for each sensor to evaluate the performance of the spectra obtained from the Cycle-GAN-based algorithm. Random and periodic noises were added to the test high-SNR spectrum and sent to the pre-trained denoising model. To determine the time efficiency of the proposed denoising algorithm, the denoising process was run several times, and the average processing time for the entire 100 spectra was calculated. With an Nvidia 4090 graphics card, the average data processing time was around 0.51 s, and the longest processing time was less than 0.68 s, indicating a significant improvement in the denoising efficiency compared to the traditional digital filter methods.

The denoising effectiveness was evaluated by comparing the denoised spectrum with the original high-SNR spectrum. Table 1 presents the SNR values for the four types of sensors before and after denoising. Since all noises were manually added at the same power level, the three FBG-based sensors provided similar SNR values ranging from 13.10 dB to 13.68 dB before applying the denoising algorithm. The simulated low-finesse FPI sensor had a spectrum shape close to a sinusoidal waveform, resulting in a relatively high SNR of 17.14 dB even before denoising. After denoising, the low-finesse FPI, regular FBG, and chirped FBG achieved an SNR of around 30 dB. With the spectrum intensity maintained at a relatively consistent rate, the noise power is reduced by 97.6%, 99.1%, and 97.9%. Similarly, the SNR of the FBG pair is relatively high for the reasons mentioned before, while its average SNR increases by 8.01 dB, corresponding to an 84.19% noise power reduction with the same signal power.

The correlation coefficient ($R^{2}$) and root mean square error (RMSE) between the denoised and original high-SNR spectra were also calculated. The average $R^{2}$ values reached 99.95%, 99.97%, 99.94%, and 99.69% for the low-finesse FPI, regular FBG, chirped FBG, and FBG pair sensors, respectively. The average RMSE of the four types of sensors also demonstrated excellent results. With the reflectivity normalized to 1, the highest RMSE, from the FBG pair, was 0.0275, while the lowest RMSE, from the regular FBG, was only 0.0067. These values indicate an outstanding ability to preserve the original spectral characteristics during the denoising procedure. Table 1 presents the results of a comparison of the sensing spectrum SNR before and after applying the denoising algorithm, along with the overall $R^{2}\;$ and RMSE between the denoised spectrum and the original high-SNR spectrum.

Wavelet thresholding (WT) and empirical mode decomposition (EMD) are two of the most used denoising techniques in fiber-optic sensing due to their advantages, such as effective noise reduction, broad applicability, and the ability to preserve the original signal characteristics. Both the WT and EMD algorithms were applied to these simulated noisy spectra to further evaluate the performance of the Cycle-GAN-based denoising algorithm in comparison to these traditional methods. Table 2 presents the results, where the first three rows show the SNRs, $R^{2}$ values, and RMSEs from the denoising spectra using the WT algorithm, while rows four to six display the corresponding values obtained using the EMD algorithm. Adjacent to each SNR, $R^{2}$ value, and RMSE, we also list the differences between these values and those obtained using the Cycle-GAN-based algorithm. These difference values are calculated by directly subtracting the values from Table 1. Hence, a negative value of SNR and $R^{2}$ or a positive value of RMSE indicates an improvement of our denoising algorithm over the traditional methods.

The SNR, $R^{2}$, and RMSE of the low-finesse FPI obtained from the WT are very close to those obtained using the Cycle-GAN-based algorithm, differing by only −0.82 dB in SNR, −0.01% in $R^{2}$, and +0.0013 in RMSE. However, when examining the denoising performance on spectra with more detail, the output differences between WT and the Cycle-GAN-based algorithm dramatically increase. In the case of the regular FBG and FBG pair sensors, most of the remaining noise from the WT is located on the sideband. The interferences in the FBG pair sensor introduce additional narrow valleys and fringe repetitions in the spectrum, resulting in an SNR of only 14.08 dB and a 97.99% $R^{2}$ after applying the WT method. The FBG pair also provides the highest RMSE, nearly double the value in Table 1. Although the regular FBG demonstrates a relatively high SNR compared to the FBG pair, it still shows an SNR difference of over 13 dB and an RMSE three times larger than the value from the Cycle-GAN-based denoising algorithm. The results from the chirped FBG show an SNR of over 23 dB and an RMSE value close to that of the regular FBG after the WT, which may be attributed to the relatively flat spectrum. The results for the denoised parameters from EMD show a similar tendency. Due to the different algorithm characteristics, EMD gives better results for FBG and chirped FBG compared to WT. Its $R^{2}$ for the chirped FBG spectrum reaches 99.94%, which is almost the same value as that obtained by our algorithm. Meanwhile, it demonstrates the worst values with the FPI and FBG pair sensor spectra. Accordingly, the comparative results indicate that the noise reduction output of the Cycle-GAN-based denoising algorithm is superior to that of both classical algorithms. The distinction is particularly evident when the original signal has a complex spectrum containing a higher proportion of high-frequency components.

**Figure 7:**
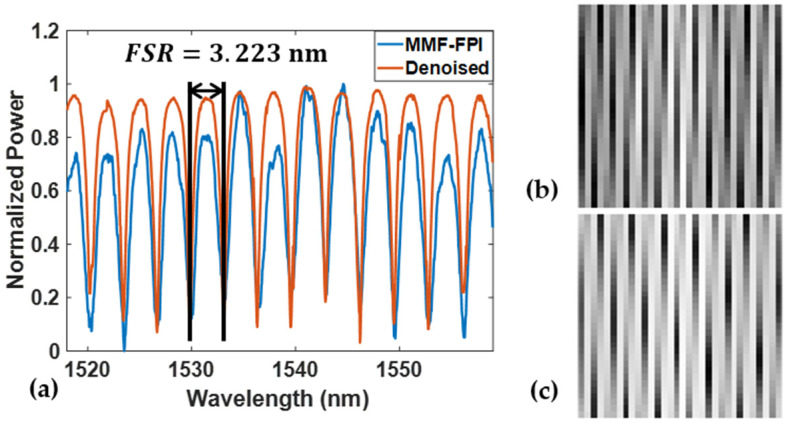
(**a**) The original MMF-FPI spectrum and its denoised output from the Cycle-GAN-based algorithm; the 2D reshaped image of (**b**) the original MMF-FPI spectrum; (**c**) the denoised spectrum.

After data collection from the setup shown in Figure 4, both SMF-FPI and MMF-FPI spectra were pre-processed and trained within the Cycle-GAN-based algorithm. Figure 7a displays the original MMF-FPI spectrum collected with MMF and its corresponding denoised spectrum processed by the algorithm. Figure 7b,c illustrate the 2D reshape images of the input and output spectra of the pre-trained denoising model. The FSR calculated for both noisy and denoised spectra is approximately 3.223 nm, indicating a close distribution of spectrum peaks and valleys. The results demonstrate excellent FSR maintenance, facilitating further wavelength analysis.

**Figure 8:**
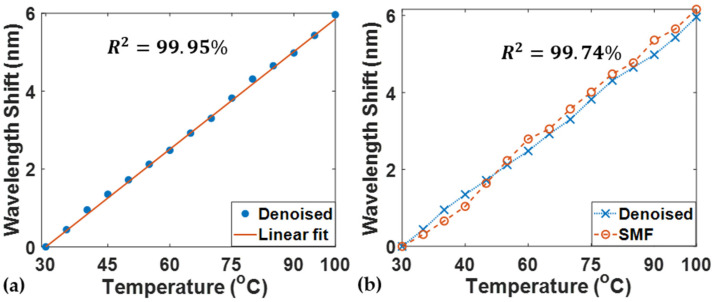
(**a**) Wavelength shift versus temperature for the denoised FPI; (**b**) wavelength shift versus temperature for SMF and denoised FPI.

The setup was also employed to perform a temperature-detection test to validate the temperature response of the denoised MMF-FPI. The spectrum was recorded for both the SMF-FPI and MMF-FPI at the same temperature. After applying the denoising algorithm, the wavelength shift was obtained by tracking one of the peaks in the spectrum. The temperature test exhibits a good linearity of the denoised FPI wavelength shift versus temperature variations, as shown in Figure 8a. The $R^{2}$ of the denoised data and its linear fit is approximately 99.95% as the temperature changes from 30 ℃ to 100 ℃ in steps of 5 ℃. The overall selected wavelength peak shift during the temperature change is 5.96 nm, indicating a temperature sensitivity of 85.1 pm/℃. This is compatible with the theoretical temperature response value of 72 pm/℃ obtained from the literature [29]. The small disparities between the denoised MMF-FPI test and the theoretical value may be attributed to the equipment and operational errors during the experiment. On the other hand, the denoised MMF-FPI exhibits a very similar wavelength shift to the data acquired from the SMF-FPI, as shown in Figure 8b. The temperature sensitivity for the SMF-FPI is 88.1 pm/℃, and the $R^{2}$ between the SMF and the denoised MMF temperature responses is 99.74%. As a result, the denoised spectrum from the Cycle-GAN-based algorithm achieves the desired temperature response linearity and sensitivity, demonstrating its superior temperature sensing capability compared with the regular SMF-FPI sensor.

**Figure 9:**
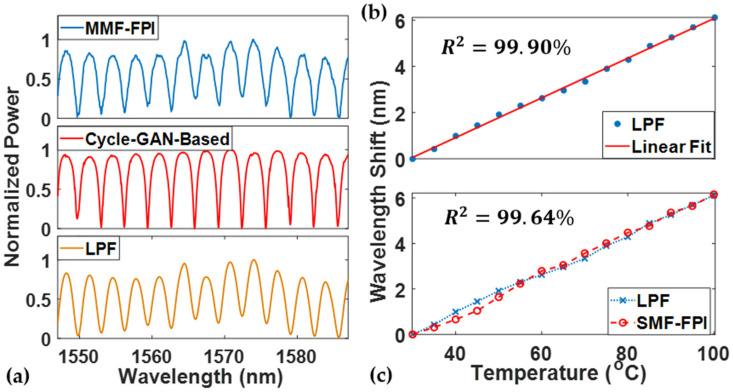
(**a**) Comparison of spectrum of MMF-FPI and noise-reduced spectra obtained via Cycle-GAN-based algorithm and low-pass filter; (**b**) wavelength shift versus temperature for MMF-FPI with LPF and its linear fit; (**c**) wavelength shift versus temperature for SMF and MMF-FPI with LPF.

A low-pass filter (LPF) was applied to process the same temperature response data to further compare the differences between the Cycle-GAN-based denoising algorithm and traditional algorithms in the testing of the MMF-FPI. Figure 9a shows the spectra of the same MMF-FPI obtained via different denoising methods. The spectrum from the LPF demonstrates a periodic wave similar to a sinusoidal function when compared to the original SMF-FPI, MMF-FPI, and denoised spectrum from the Cycle-GAN-based algorithm. Please note that the silicon wafer has a refractive index of 3.48 at the wavelength around 1550 nm, corresponding to about 30% reflectivity with air. Therefore, the spectrum processed by the LPF loses some characteristics of the high-finesse FPI, whereas these features are preserved in the spectrum obtained via the Cycle-GAN-based approach.

## 5. Conclusions

This paper presents a Cycle-GAN-based algorithm for denoising fiber-optic sensors. By reshaping the sensing spectra into a 2D format and utilizing them as images for denoising training, the model learns the characteristics of both high-SNR and low-SNR spectra. The pre-trained model can effectively eliminate the noise from the low-SNR sensing spectra while preserving the features of high-SNR spectra. In the simulation involving different types of sensor spectra, our denoising algorithm effectively addressed both the random and periodic noise, demonstrating outstanding performance in restoring the low-SNR spectrum to a high-SNR level, with improved output SNR, $R^{2}$, and RMSE compared to the traditional denoising methods. The experimental tests reveal that the denoised MMF-FPI achieves sensing capability comparable to that achieved via SMF-FPI. The results demonstrate the excellent maintenance capability to spectra features such as FSR, wavelength, and spectra shape.

In conclusion, our approach significantly improves the accuracy, generalizability, and efficiency in noise reduction of fiber-optic sensing. The exceptional denoising performance across different sensor types and noise profiles indicates a substantial improvement in fiber sensing system. The outstanding modal noise reduction capability exhibits the potentially enhanced practicality of specialty fiber sensors, such as sapphire and other multimode fibers, in meeting specific application demands.

## Figures and Tables

**Table 1:** Comparison of SNR of noisy spectra and denoised spectra, and the denoised $R^{2}$ values and RMSE of the four different types of sensors.

Sensor Type	FPI	FBG	Chirped FBG	FBG Pair
Noisy SNR (dB)	17.14	13.10	13.17	13.68
Denoised SNR (dB)	33.46	33.19	29.97	21.69
$\mathrm{Denoised}\;R^{2}$ (%)	99.95	99.97	99.95	99.70
Denoised RMSE	0.0133	0.0067	0.0149	0.0275

**Table 2:** Comparison of noise reduction results: SNRs, $R^{2}$ values, and RMSEs from the WT and EMD algorithms against to those from the Cycle-GAN-based denoising algorithm.

Sensor Type	FPI	FBG	Chirped FBG	FBG Pair
WT SNR (dB)	32.64 (−0.82)	19.48 (−13.71)	23.10 (−6.87)	14.08 (−7.61)
$\mathrm{WT}\;R^{2}$ (%)	99.94 (−0.01)	99.77 (−0.20)	99.88 (−0.07)	97.99 (−1.71)
WT RMSE	0.0146 (+0.0013)	0.0329 (+0.0262)	0.0328 (+0.0179)	0.0624 (+0.0349)
EMD SNR (dB)	30.73 (−2.73)	26.66 (−6.53)	23.96 (−6.01)	13.59 (−8.10)
$\mathrm{EMD}\;R^{2}$ (%)	99.84 (−0.11)	99.90 (−0.07)	99.94 (−0.01)	97.66 (−2.04)
EMD RMSE	0.0197 (+0.0064)	0.0295 (+0.0228)	0.0298 (+0.0149)	0.0659 (+0.0384)

## Data Availability

The data presented in this study are available on request from the corresponding author due to restrictions.
